# Protists as main indicators and determinants of plant performance

**DOI:** 10.1186/s40168-021-01025-w

**Published:** 2021-03-20

**Authors:** Sai Guo, Wu Xiong, Xinnan Hang, Zhilei Gao, Zixuan Jiao, Hongjun Liu, Yani Mo, Nan Zhang, George A. Kowalchuk, Rong Li, Qirong Shen, Stefan Geisen

**Affiliations:** 1 Jiangsu Provincial Key Lab of Solid Organic Waste Utilization, Jiangsu Collaborative Innovation Center of Solid Organic Wastes, Educational Ministry Engineering Center of Resource-saving fertilizers, Nanjing Agricultural University, Nanjing, 210095 PR China; 2 The Key Laboratory of Plant Immunity, Nanjing Agricultural University, Nanjing, 210095 PR China; 3 Ecology and Biodiversity Group, Department of Biology, Institute of Environmental Biology, Utrecht University, Padualaan 8, Utrecht, 3584 CH The Netherlands; 4 Laboratory of Nematology, Wageningen University, Wageningen, 6700 AA The Netherlands; 5 Netherlands Department of Terrestrial Ecology, Netherlands Institute for Ecology, (NIOO-KNAW), Wageningen, 6708 PB The Netherlands

**Keywords:** Soil protist community, Soil management, Organic fertilizers, Crop yield enhancement

## Abstract

**Background:**

Microbiomes play vital roles in plant health and performance, and the development of plant beneficial microbiomes can be steered by organic fertilizer inputs. Especially well-studied are fertilizer-induced changes on bacteria and fungi and how changes in these groups alter plant performance. However, impacts on protist communities, including their trophic interactions within the microbiome and consequences on plant performance remain largely unknown. Here, we tracked the entire microbiome, including bacteria, fungi, and protists, over six growing seasons of cucumber under different fertilization regimes (conventional, organic, and *Trichoderma* bio-organic fertilization) and linked microbial data to plant yield to identify plant growth-promoting microbes.

**Results:**

Yields were higher in the (bio-)organic fertilization treatments. Soil abiotic conditions were altered by the fertilization regime, with the prominent effects coming from the (bio-)organic fertilization treatments. Those treatments also led to the pronounced shifts in protistan communities, especially microbivorous cercozoan protists. We found positive correlations of these protists with plant yield and the density of potentially plant-beneficial microorganisms. We further explored the mechanistic ramifications of these relationships via greenhouse experiments, showing that cercozoan protists can positively impact plant growth, potentially via interactions with plant-beneficial microorganisms including *Trichoderma,* the biological agent delivered by the bio-fertilizer.

**Conclusions:**

We show that protists may play central roles in stimulating plant performance through microbiome interactions. Future agricultural practices might aim to specifically enhance plant beneficial protists or apply those protists as novel, sustainable biofertilizers.

Video abstract

**Supplementary Information:**

The online version contains supplementary material available at 10.1186/s40168-021-01025-w.

## Background

Soil is the basis for crop production [1, 2] by providing water, nutrients, and the growth matrix for plants [3, 4]. However, space for agricultural use of soils is limited, as other human needs and natural resources compete for this space [5, 6]. Given this limitation for space, intensive agricultural management systems, including continuously growing the same crop, have been developed to help to meet the increasing food demands of a growing human population [7–9]. However, such continuous cropping systems commonly suffer from a buildup of soil-borne plant pathogens [10], an imbalance in nutrient availability [8], and a reduction of soil fertility [11–13], which together can negatively affect yield [14–16]. The application of chemical fertilizers and pesticides used in conventional agricultural practices do help to sustain high crop yields, but the overuse of these agrochemicals can lead to severe environmental problems [7, 17]. Organic soil management might provide a solution to mitigate yield reductions by increasing soil quality and nutrients and reducing soil-borne diseases, with far fewer negative environmental impacts than induced by conventional agricultural practices [18–20].

Bacteria and fungi, which represent the most studied soil microbial groups, are known to be impacted by fertilization regimes [11, 18, 21]. Shifts in these communities can be linked to plant productivity and health status through direct mutualistic or pathogenic effects on plants and indirectly by competing with plant-associated microorganisms [22–24]. However, bacteria and fungi are embedded in complex soil food webs in which predation can structure their community composition and functioning [25, 26]. The main consumers of soil bacteria and fungi are protists, the most diverse and abundant soil eukaryotes [27]. Protists also display phototrophic, animal parasitic, and plant pathogenic lifestyles [27]. Protistan communities are influenced by numerous environmental factors and soil properties [28, 29], and they have long been proposed as sensitive bio-indicators of soil quality [30]. Logically, differences in fertilization practices affect protistan communities [31], potentially even more than bacterial and fungal communities [32]. Protistan communities might also best predict and potentially control plant health in the presence of soil-borne pathogens [33]. Yet, the functional link between fertilization-induced shifts in protistan communities to their microbial prey and crop yield remains essentially unknown.

To better understand the impact of different fertilization practices on microbiome composition and functioning, we studied the entire soil microbiome, with a particular emphasis on protists, in continuously planted cucumber soils for six growing seasons. Fertilizer treatments consisted of conventional, organic, and bio-organic (organic fertilizer with the addition of *Trichoderma* fungus) fertilization, in addition to a no-fertilizer control. We then linked microbiome taxonomic and functional shifts to crop yield and performed subsequent greenhouse experiments to validate the functional importance of key protists with and without the presence of other (plant-beneficial) microorganisms on plant performance. We hypothesized that greater yield would be realized in both organic fertilization treatments, with protists representing the microbial group most responsive to different fertilization practices. We further hypothesized that crop yield could be at least partly explained by protist community structure and the relative abundance of specific protistan taxa.

## Results

### Crop yield and yield contribution of soil microbiome communities

**Fig. 1:**
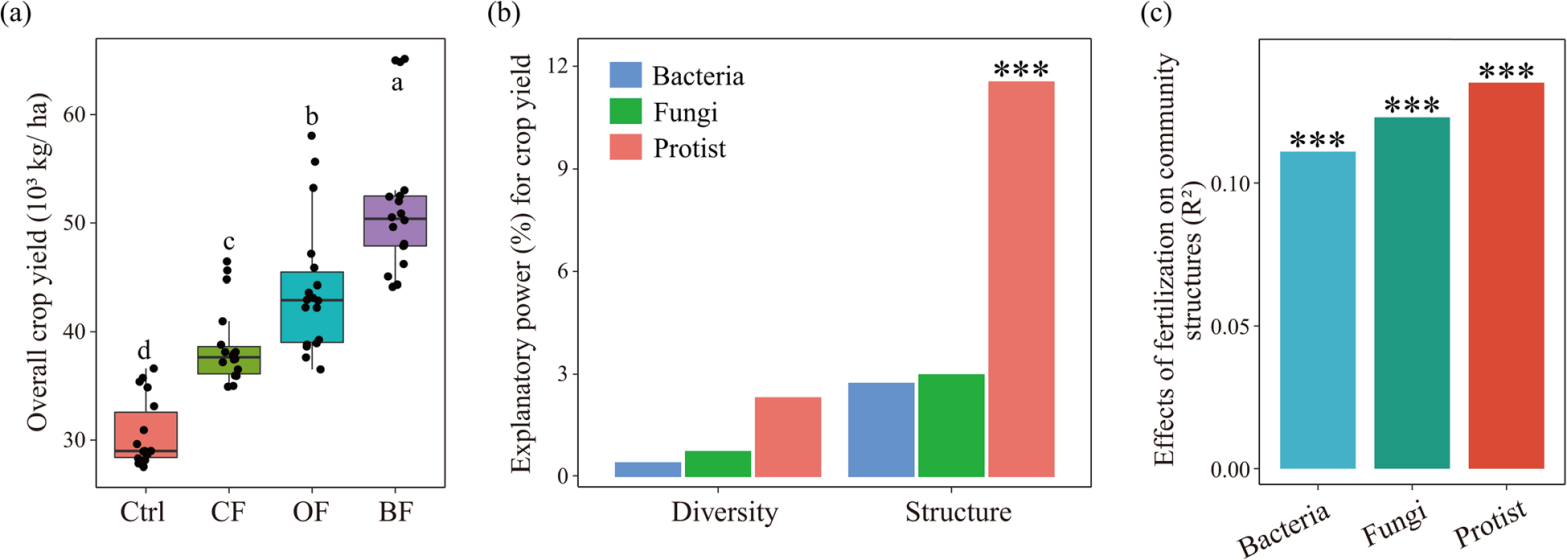
Overall effects of different fertilization practices on crop yield (**a**). The relative importance of bacterial, fungal and protistan diversity and community structure for crop yield (**b**). The effects of fertilization regime on bacterial, fungal and protistan community structure (**c**). In panel (**a**), bars with different letters indicate significant differences as defined by Tukey’s HSD test (*P* < 0.05). Ctrl: soil without fertilizer amendment; CF: soil amended with chemical fertilizer; OF: soil amended with organic fertilizer; BF: soil amended with bio-organic fertilizer. In panels (**b**) and (**c**), ***indicates *P* < 0.001. Statistical significance for explanatory power was calculated by multiple regression using linear models. Effect value (*R*^2^) and statistical significance were calculated by PERMANOVA analysis

The application of all fertilizers increased crop yield in all six continuous cropping seasons in comparison with the control (*P* < 0.05; Fig. S1a). Crop yields in OF and BF were higher than that of CF in all seasons combined (*P* < 0.05; Fig. 1a) and within each season (Fig. S1a). Protistan community structure was the best microbial parameter among the selected microbial indices with respect to explaining crop yield across all treatments (*P* < 0.05), explaining 11.56 % of the observed variation (Fig. 1b). In contrast, neither the bacterial or fungal community structure, nor the diversity of any microbial group was significantly linked to yield (*P* > 0.05; Fig. 1b).

### Microbial diversity and community structure

Fertilization regimes affected the community structure of all microbial groups, with the strongest impact on protists (protists: *R*^2^ = 0.135, *P* = 0.001; bacteria: *R*^2^ = 0.111, *P* = 0.001; fungi: *R*^2^ = 0.123, *P* = 0.001) (Fig. 1c and Fig. S1b and Table S2). The alpha diversity of protists was marginally affected by fertilization (*F* = 3.944, *P* = 0.053), while alpha diversity of both bacteria (*F* = 0.881, *P* = 0.491) and fungi (*F* = 2.166, *P* = 0.170) was not altered (Table S3). The only significant difference for alpha diversity between treatments was a higher protistan diversity in OF compared with CF (*P* < 0.05; Fig. S1c). A higher variation of protistan diversity in all fertilization treatments was observed than that in control treatment (Fig. S1d). As protists responded most strongly to fertilization and given their link with yield, we focused subsequent analyses on protists.

### Underlying drivers of protistan taxonomic and functional composition and links with yield

**Fig. 2:**
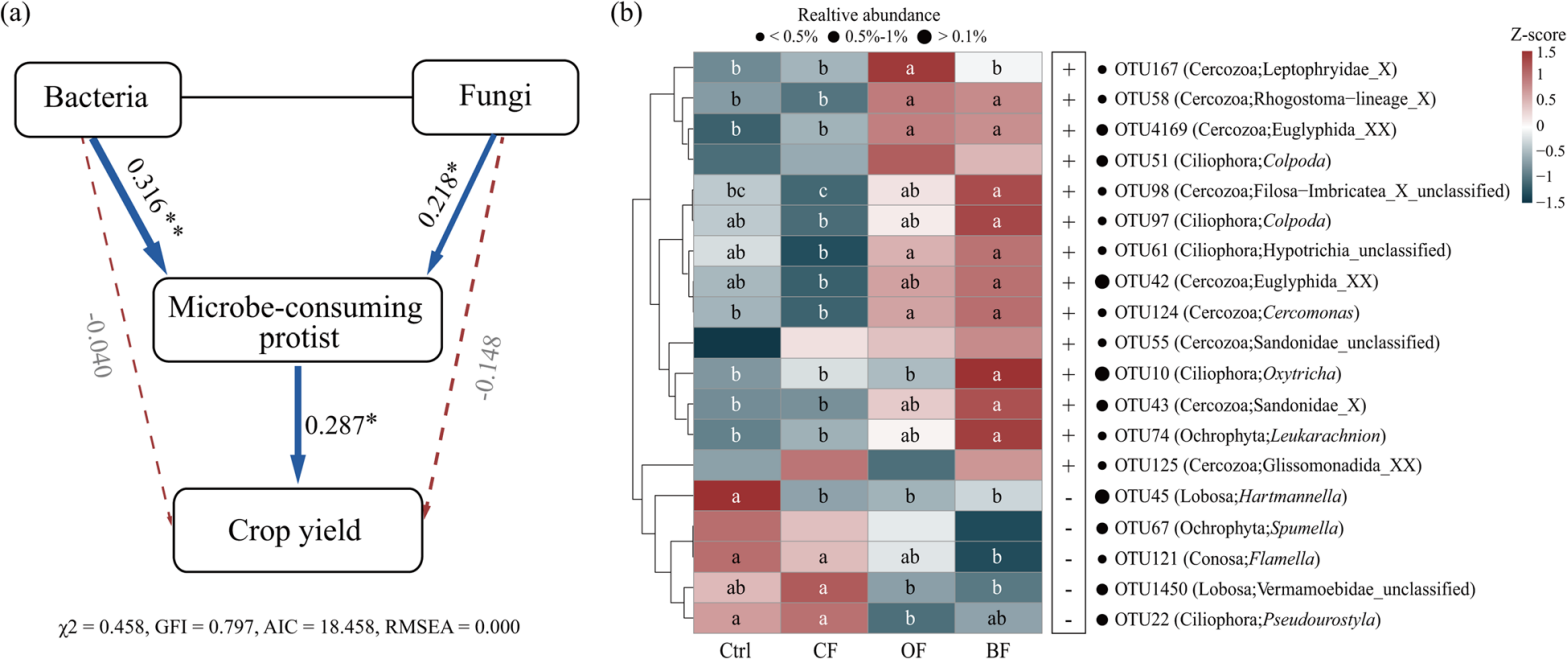
Structural equation model (SEM) illustrating the links between bacterial and fungal abundances and the relative abundance of microbe-consuming protists on crop yield (**a**). Heatmap illustrating the relative abundance of microbe-consuming protistan OTUs associated with crop yield in all treatments (**b**). In panel (**a**), *indicates *P* < 0.05; **indicates *P* < 0.01. Solid and dashed lines represent significant and non-significant relationships, respectively, with blue arrows depicting positive relationships and red arrows negative relationships. Numbers associated with lines indicate path coefficients, and line width is proportional to the effect size. The low chis-square (*χ*2 = 0.458), high goodness-of-fit index (GFI = 0.797), low Akaike information criteria (AIC = 18.458), and low root mean-square errors of approximation (RMSEA = 0.000) indicate that our data matches our hypothetical model. In panel (**b**), the colour key relates the heatmap colours to the standard score (*z*-score). Plus signs indicate positive, and minus signs negative correlations between OTUs and crop yield. Different letters indicate significant differences as defined by Tukey's HSD test (*P* < 0.05). Circles are proportional to the average relative abundance of each group across all samples

Among protistan functional groups, only microbe-consuming protists positively correlated with yield (*P* < 0.05; Table S4). Compared with the control, the relative abundance of microbe-consuming protists increased by 4.28 % in BF (*P* < 0.05), while there were no significant differences across the other treatments (Fig. S1e). The relative abundance of microbe-consuming protists significantly decreased over time only in CF (*P* < 0.05; Fig. S1f). In addition, we detected lower variations of the relative abundance of microbe-consuming protists in (bio-)organic fertilization treatments (organic and *Trichoderma* bio-organic fertilization) compared with chemical fertilization treatment (Fig. S1f). We performed SEM to further identify potential links between yield with microbe-consuming protists, bacteria, and fungi. This analysis indicated that microbe-consuming protists were directly linked to yield (*P* < 0.05; path coefficient = 0.287) through interactions with bacteria (*P* < 0.01; path coefficient = 0.316) and fungi (*P* < 0.05; path coefficient = 0.218) (Fig. 2a). Analyses of abiotic factors potentially underlying fertilization-induced changes of microbe-consuming protists revealed soil pH as the major factor among the eight measured soil physicochemical properties (*P* < 0.05; Fig. S2a, b and c).

Fourteen microbe-consuming protistan OTUs positively correlated with crop yield, including nine cercozoan, four ciliophoran, and one ochrophytan taxa (Fig. 2b). Of these, the cercozoan OTU124 (Cercozoa, *Cercomonas*), OTU4169 (Cercozoa, Euglyphida_XX), and OTU58 (Cercozoa, Rhogostoma−lineage_X) showed significantly higher relative abundance in OF and BF as compared with the Control and CF treatments (*P* < 0.05; Fig. 2b). Spearman’s correlation analysis, used to further identify potential links between these microbe-consuming protists and bacteria or fungi, indicated that OTU124 was positively correlated with *Trichoderma* (F_OTU2929) and *Aspergillus* (F_OTU14), OTU4169 with *Pseudomonas* (B_OTU1, B_OTU1938) and *Aspergillus* (F_OTU14, F_OTU23), while OTU58 was negatively correlated with *Fusarium* (F_OTU3) (Fig. S2d).

### Plant growth promotion capability of microbe-consuming protist isolates

**Fig. 3:**
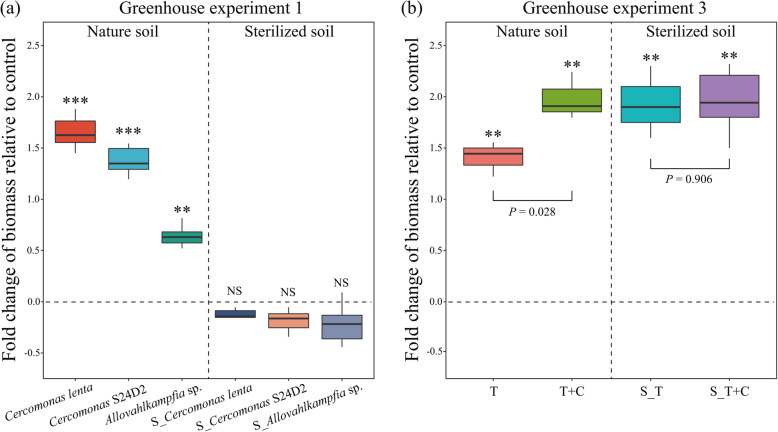
Fold change of cucumber biomass relative to control in treatments with inoculation of different microbe-consuming protists in a first greenhouse experiment (**a**) (for the confirmatory second greenhouse experiment see Fig. S3). Cucumber biomass of the third greenhouse experiment testing plant growth promotion capability of *Trichoderma* stimulated by the inoculation of microbe-consuming protist (**b**). In panel (**a**), control indicates that no protists were inoculated; S_ indicates that protists were inoculated into sterilized soil. In panel (**b**), control indicates that no microbe was inoculated; S_ indicates that protists and fungi were inoculated into sterilized soil. T: *Trichoderma*; T+C: *Trichoderma*+*Cercomonas lenta*. Asterisks indicate significant difference of cucumber biomass in treatments compared with control (Student’s *t* test, ***indicates *P* < 0.001; **indicates *P* < 0.01; NS indicates not significant). The *P* values indicate significance between T and T+C or S_T and S_T+C under Student’s *t* test

Our first greenhouse experiment aimed at mechanistically understanding the observed links between protistan communities and crop yield in the field study. We found that all microbe-consuming protists increased cucumber biomass as compared with the control in natural soils (*P* < 0.05), but this was not the case in sterilized soils (Fig. 3a). The strongest positive effect on plant performance was induced by the two cercozoan taxa *Cercomonas lenta* (an increase of 165%) and *Cercomonas* S24D2 (an increase of 138%) (Fig. 3a). The non-cercozoan microbe-consuming *Allovahlkampfia* sp. also increased plant biomass compared with the control (64%), but less than *Cercomonas lenta* and *Cercomonas* S24D2 (Fig. 3a). The positive effects of the two cercozoan species, *Cercomonas lenta* and *Cercomonas* S24D2, on plant performance, were confirmed in a second greenhouse experiment, showing increases of plant biomasses by 101% and 79% compared with control, respectively (Fig. S3).

The third greenhouse experiment was designed to explore potential interactions between microbe-consuming protists and *Trichoderma* (the biological agent added to organic fertilizers in the BF treatment). In sterilized soil, the strongest positive effects on plant performance were observed when *Trichoderma* was inoculated without protists (S_T; 193% increase to control) and in Trichoderma+protist inoculations (S_T+C; 197%) (*P* < 0.05; Fig. 3b). In natural soil, T+C significantly increased the plant biomass when compared with T (*P* = 0.028), while both T and T+C increased plant biomass by 140% and 196% compared with control, respectively (*P* < 0.05; Fig. 3b).

## Discussion

We found that protists, especially microbe-consumers, were the microorganisms most strongly affected by fertilization practices. Protistan communities as well as specific protistan taxa were mostly strongly explaining crop yield variation via interactions with bacterial and fungal communities. Our findings suggest that the protistan community plays a previously neglected role in determining plant performance, which can be at least as important as bacterial and fungal communities that are typically targeted in soil and rhizosphere microbiome studies. This extends previous studies that show protists to be the most sensitive microbial group to fertilization [32] and the best microbial community determinant of plant health [33]. Indeed, we here show evidence for a potential positive link between protistan communities and plant yield, a finding that we support with findings from greenhouse studies that show protist-induced plant growth increases. The importance of different protistan communities and species might be explained by species-specific protistan feeding differences [34, 35] that might specifically shape a plant growth-promoting microbiome [26].

Modification in abiotic soil parameters through fertilization, particularly the associated soil acidification, was a driver of changes in protistan diversity, community structure, and functional composition, which confirms the importance of abiotic parameters as global and local determinants of protistan communities [28]. Indeed, our study suggests that pH might be of similar importance in structuring protists at the local scale as soil moisture at both local and global scales [29, 36]. Especially phagotrophic microbe-consumers, as the dominant soil protistan functional group [36], responded strongly to changes in soil physicochemical properties, potentially partly due to co-occurring changes in bacterial and fungal prey communities. The addition of *Trichoderma* to organic fertilizer (BF treatment) also affected protistan communities, confirming that microbial introductions can change protistan communities [37]. Our results also show the tight link between communities of bacteria and protists as repeatedly shown before [38], highlighting protists as key predators of soil bacteria [27, 39]. We also show links between fungi and protists that might be linked to their emerging role as important fungivores [40]. This ancient co-evolutionary predator-prey relationship likely resulted in the abovementioned species-specific feeding differences as well as highly sophisticated antagonism mechanisms, in which bacteria and fungi defend against predators [26, 41].

The strong link between organic fertilization enriched microbe-consuming protists, particularly cercozoan taxa, and plant yield in the field might be explained by a combination of effects including enhanced nutrient turnover, a promotion of plant-beneficial microorganisms and a manipulation of the plant hormonal balance [27, 42, 43]. Indeed, microbe-consuming protists are suggested to enhance plant performance by preying on plant-detrimental microbes or increasing the performance of plant growth-promoting microorganisms through predation on their competitors [26, 42]. Our greenhouse experiments confirmed the positive plant growth-promoting effect of microbe-consuming protists (*Cercomonas lenta*; *Cercomonas* S24D2; *Allovahlkampfia* sp.), particularly of Cercozoa, globally one of the most abundant and functionally important soil protist group [36, 44]. These cercozoan taxa observed here might increase plant yield through negative links with plant pathogens (*Fusarium*) and positive links with plant-beneficial microorganisms (*Pseudomonas*, *Trichoderma*, and *Aspergillus*), a link that we highlight in our greenhouse experiments (Fig. 3b).

Our third greenhouse experiment provided direct evidence for the functional importance of *Trichoderma* in enhancing plant performance [45]. The stronger positive effect on plant performance of *Trichoderma* in sterilized than in natural soils suggests that competitive interactions with other soil organisms reduce the positive effect of *Trichoderma* [46, 47], while other indigenous soil-borne pathogens might also directly inhibit plant performance [33]. No significant difference was observed between “S_T” and “S_T+C,” indicating that *Cercomonas lenta* might not directly interact with *Trichoderma*. The stronger positive effect on plant performance was induced in “T+C” compared with the “T,” suggesting that *Cercomonas lenta* may increase the competitive ability of *Trichoderma* in natural soil that increases its positive effects on plant performance. Those results provide a novel viewpoint to manipulate the soil microbiome by combining protists with plant-beneficial fungi, which might help increase agro-ecosystem functions in a sustainable way.

## Conclusions

We show that protistan communities, and in particular microbe-consuming taxa, are important microbial determinants of crop yield via their specific enhancement of plant-beneficial microorganisms. Microbe-consuming protists represented the microbial group most strongly affected by fertilization. Therefore, these protists may represent a powerful tool for targeted and environmentally friendly microbiome manipulation to increase crop yield. We highlight the need to include protists in studies seeking to evaluate the impacts and mechanisms involved in bio-organic fertilizer applications, and future research should evaluate the use of protists as bioadditives for the development of new strategies for sustainable protist-supported plant growth promotion.

## Methods

### Field description, experimental design, and soil sample collection

The long-term continuous cropping field was initiated in March 2014 and is located at the Nanjing Institute of Vegetable Science, Jiangsu province, China (31° 43′ N, 118° 46′ E). It is situated in a subtropical monsoon climate area with an annual average temperature of 15.4 °C and an average precipitation of 1106 mm.

Cucumber was the crop that was continuously planted from March 2014 through October 2016, and treatments were established in a randomized complete block design with three replicates for each fertilizer treatment. Briefly, the four fertilization treatments were: Ctrl, soil amended with no fertilizer; CF, soil amended with chemical fertilizer; OF, soil amended with organic fertilizer and BF, soil amended with bio-organic fertilizer. A detailed fertilization scheme (average amount of six seasons) is shown in Table S1. In brief, the organic fertilizer is a mixture of compounds including liquid amino acids from animal carcasses and chicken manure compost at a ratio of 1:5 (v/w). The bio-organic fertilizer is composed of organic fertilizer with approximately 5.0×10^7^ CFU dry weight of *Trichoderma guizhouense* NJAU 4742 g^−1^ dry fertilizer. Here, the strain *Trichoderma guizhouense* NJAU 4742 has a strong growth-promoting ability and is a widely used commercial biological agent in China [48, 49]. A rotary tiller was used to apply different fertilizers before cucumber planting, and all treatments were managed in the same way throughout the experiment. Soil samples were collected and crop yield was detected during full bearing period in June 2014 (subsequently termed “Crop 1”), October 2014 (“Crop 2”), June 2015 (“Crop 3”), October 2015 (“Crop 4”), June 2016 (“Crop 5”) and October 2016 (“Crop 6”). For each replicate, a composite soil sample was generated from 8 random soil cores taken to a depth of 5 cm using a 25-mm soil auger. Samples were thoroughly homogenized and sieved through a 2-mm mesh-size sieve. One portion of each sample was air-dried for chemical analyses, and the other portion was stored at −80 °C for subsequent DNA extraction.

### Analysis of soil physicochemical properties

Soil pH was measured in a 1:5 soil/water suspension with a glass electrode. Soil organic matter (SOM) was measured using the K_2_Cr_2_O_7_-H_2_SO_4_ oxidation-reduction colorimetric method [32]. Total nitrogen (TN) was analyzed by an elemental analyzer (multi EA® 5000, Analytik Jena, Germany). Total phosphorus (TP) was determined by the molybdenum blue method after wet digestion with H_2_SO_4_+HClO_4_ [50]. Total potassium (TK) was determined after digesting the sample with HNO_3_+HClO_4_ by flame photometry [51]. Available nitrogen (AN) was measured with the alkaline-hydrolysable diffusion method [52]. Available phosphorus (AP) in the soil was extracted with sodium bicarbonate and determined using the molybdenum blue method. Available potassium (AK) in the soil was extracted with ammonium acetate and determined by flame photometry [53].

### DNA extraction, real-time PCR assay, and Illumina MiSeq sequencing

For each composite soil sample, total DNA was extracted from 10 g of soil using the DNeasy® Power max® Soil Kit (Qiagen, Germany), according to the manufacturer’s instructions. Genomic DNA concentration and purity were measured using a NanoDrop ND-2000 (NanoDrop Technologies, Wilmington, DE) spectrophotometer. Abundances of bacteria and fungi as estimated by 16S rRNA gene and ITS region, respectively. Copy numbers were determined with the primer combinations Eub338F/Eub518R and ITS1f/5.8s, respectively, following established protocols [18] on a qTOWER Real-Time PCR System (Analytik Jena, Germany). We set up 20-μl reaction mixtures containing 10 μl of the SYBR®Premix Ex Taq™ (TaKaRa, Japan), 0.4 μl of each primer (10 μM), 2 μl of template DNA, and 7.2 μl of ddH_2_O. Standard curves were generated by using 10-fold serial dilutions of a plasmid containing a full-length copy of the 16S rRNA gene from *Escherichia coli* or the 18S rRNA gene from *Saccharomyces cerevisiae*. Thermal-cycling conditions for each sample were run as follows: 30 s at 95 °C for initial denaturation, 40 cycles of 5 s at 95 °C, and 34 s at 60 °C, and the results were expressed as log copy numbers g^−1^ dry soil.

Prokaryote-, fungal-, and eukaryote-wide primers sets were used for high-throughput Illumina MiSeq sequencing: 515F (5′-GTGCCAGCMGCCGCGGTAA-3′) and 806R (5′-GGACTACHVGGGTWTCTAAT-3′) to amplify prokaryotic 16S rRNA gene V4 regions [54], ITS1F (5′-CTTGGTCATTTAGAGGAAGTAA-3′) and ITS2 (5′-GCTGCGTTCTTCATCGATGC-3′) to amplify fungal ITS1 regions [55, 56], and V4_1f (5′-CCAGCASCYGCGGTAATWCC-3′) and TAReukREV3 (5′-ACTTTCGTTCTTGATYRA-3′) to amplify eukaryotic 18S rRNA gene V4 regions [57]. These primer pairs were modified for sequencing by adding the forward Illumina Nextera adapter, a two base pair “linker” sequence, and a unique 7-bp barcode sequence at the 5′ end of the forward primer, and the appropriate reverse Illumina Nextera adapter and linker sequence to the 5′ end of the reverse primer. PCR amplification was performed in a 25 μl volume: 5 μl of 5 × reaction buffer, 5 μl of 5 × GC buffer, 2 μl dNTPs (2.5 mM), 1 μl of each primer (10 uM), 0.25 μl of high-fidelity DNA polymerase, 2 μl of DNA template and 8.75 μl of ddH_2_O. The PCR thermal cycling conditions were performed with the following temperature regime: initial denaturation (98 °C for 2 min), followed by 30 cycles of denaturation (98 °C for 15 s), annealing (55 °C for 30 s), extension (72 °C for 30 s), and a final extension (72 °C for 5 min). Each sample was amplified in triplicate, pooled in equimolar concentrations of 20 ng μl^−1^, and then purified with a PCR Purification Kit (Axygen Bio, USA). Paired-end sequencing was performed on an Illumina MiSeq sequencer at Personal Biotechnology Co., Ltd (Shanghai, China). All raw sequence data are deposited in the NCBI Sequence Read Archive (SRA) under the BioProject PRJNA599073.

### Bioinformatic analyses

The bacterial, fungal, and protistan raw sequences were processed according to previously established protocols [58, 59]. Briefly, low-quality sequences and singletons were removed. After that, the remaining sequences were assigned to OTUs at a 97% similarity threshold, and chimeras were filtered using UCHIME [60]. Finally, representative sequences for bacterial and fungal OTUs were classified using the RDP classifier against the RDP Bacterial 16S rRNA gene database and the UNITE Fungal ITS database, respectively [61]. 18S rRNA gene sequences were matched against the Protist Ribosomal Reference database (PR2) [62]. We discarded OTUs assigned as Rhodophyta, Streptophyta, Metazoa, Fungi, and unclassified Opisthokonta sequences to generate a protistan-focused OTU table for subsequent analyses. We further assigned taxonomic protistan OTUs into different functional groups (microbe-consuming, phototrophic, plant pathogenic, animal parasitic, and saprophytic), according to their feeding mode based [37, 44, 63]. Relative abundances of each protistan taxonomic and functional group in relation to total protistan reads were used for later analyses.

### Pot experiments to test the effects of microbe-consuming protistan isolates on cucumber growth

Firstly, we tested the effects of microbe-consuming protists on cucumber growth in the first and second greenhouse experiments. We used two *Cercomonas* cultures (*Cercomonas lenta* strain ECO-P-01 and *Cercomonas* sp. strain S24D2) as these were indicative for high plant growth. Furthermore, we used another protist, *Allovahlkampfia* sp. strain NL10 as a control to test how protists not linked to high yield affect plant growth. Secondly, microbe-consuming protists were tested for their stimulatory effect on the plant growth promotion capacity of *Trichoderma* in the third greenhouse experiment. Experimental soil was collected from the control treatment in the above-mentioned long-term field experiment site. Soils were mixed and passed through a 2-mm sieve. One portion of the soil was stored at room temperature, and the other sterilized by gamma irradiation (60 KGy). Cucumber seeds were sterilized by immersion in sodium hypochlorite (3%) for 30 seconds and ethanol (75%) for 2 minutes, followed by 20-fold immersion in sterile water. Pot experiments were constructed using polypropylene pots filled with 100 g dry soil and hydrated with sterile water and cultivated in a Plant Illuminated Incubator (daytime: 12 hours and 28 °C, night: 12 hours and 25 °C, all-day average humidity of 50 %) with periodic randomization throughout the experiment.

1. Greenhouse experiments 1 and 2: Effects of microbe-consuming protists on cucumber growth

Seven inoculation treatments were designed in the first greenhouse experiment as follows: (1) *Cercomonas lenta*: *Cercomonas lenta* strain ECO-P-01 in 5 ml PAS buffer (Page’s Amoeba Saline: 120 mg NaCl, 4 mg MgSO_4_·7H_2_O, 4 mg CaCl_2_·2H_2_O, 142 mg Na_2_HPO_4_, and 136 mg KH_2_PO_4_ in 1 liter of distilled water) [64] was inoculated into natural soil, (2) *Cercomonas* S24D2: *Cercomonas* sp. strain S24D2 with 5 ml PAS buffer was inoculated into natural soil, (3) *Allovahlkampfia* sp.: *Allovahlkampfia* sp. strain NL10 in 5 ml PAS buffer was inoculated into natural soil, (4) S_ *Cercomonas lenta*: *Cercomonas lenta* strain ECO-P-01 in 5 ml PAS buffer was inoculated into sterilized natural soil, (5) S_ *Cercomonas* S24D2: *Cercomonas* sp. strain S24D2 in 5 ml PAS buffer was inoculated into sterilized natural soil, (6) S_ *Allovahlkampfia* sp.: *Allovahlkampfia* sp. strain NL10 in 5 ml PAS buffer was inoculated into sterilized natural soil, and (7) Control, 5 ml PAS buffer was added into natural soil. A follow-up experiment was conducted in the same way as described above, but focusing on *Cercomonas lenta* and *Cercomonas* S24D2 and a non-protist Control to validate the effects observed in the first experiment. Protists were inoculated by adding 1.0 × 10^4^ protistan cells g^−1^ dry soil in 5 ml PAS buffer in sterilized and natural soils and added sterile water to 40% soil moisture. The three protist strains used for experimentation, *Cercomonas* sp. strain S24D2, *Cercomonas lenta* ECO-P-01, and *Allovahlkampfia* sp. strain NL10, were isolated and identified by [65].

2) Greenhouse experiment 3: Testing interactions between protists and plant beneficial *Trichoderma* on the resident microbiome and plant growth

*Trichoderma* sp*.* isolates were randomly selected after recovery from soil collected from the BF treatment in the field experiment. In brief, 10 g of soil was suspended in a 250-ml Erlenmeyer flask containing 90 ml of sterile distilled water. After stirring at 180 rpm for 40 min, serial dilutions were spread onto plates containing Martin’s semi-selective medium (per liter: 18 g agar, 10 g dextrose, 0.5 g MgSO4, 0.5 g peptone, 0.5 g beef extract, 0.05 g bengal pink and 0.3 g chloramphenicol), and plates were incubated at 28 ± 1°C for 7 days. Colonies with typical *Trichoderma* morphology were transferred to potato-dextrose agar (PDA), incubate at 28 ± 1 °C, and identified based on the ITS region sequence analysis as described previously [66]. Six treatments in sterilized and non-sterilized soils were implemented with controls consisting of 5 ml PAS buffer added to sterilized (S_Control) and alive (Control) soils. *Trichoderma* treatments were created by adding 1.0 × 10^4^ *Trichoderma* spores g^−1^ dry soil in 5 ml PAS buffer in sterilized (S_T) and non-sterilized (T) soils. *Trichoderma*+*Cercomonas lenta* treatments consisted of 1.0×10^4^ *Trichoderma* spores g^−1^ dry soil and 1.0 × 10^4^ *Cercomonas lenta* strain ECO-P-01 cells g^−1^ dry soil in 5 ml of PAS buffer in sterilized (S_T+C) and non-sterilized (T+C) soils. All solutions were evenly inoculated into soils, and sterile water to 40% soil moisture was added.

Plant samples were collected after two weeks for experiments 1 and 3 and after 1 week for experiment 2. Shoots were oven-dried at 65 °C for 5 days before measuring dry biomass.

### Statistical analyses

We estimated bacterial, fungal, and protistan α-diversity using non-parametric Shannon indexes. For β-diversity, principal coordinate analysis (PCoA) based on the unweighted UniFrac distance was used to explore differences of bacterial, fungal, and protistan community structures across all soil samples. Linear mixed models (LMM) were performed to assess the effects of fertilization and crop season on the diversity of the soil microbiome, with the plot position serial number (i _ j, are the row and column number of the plot, respectively) as a random effect in “lme4” and “lmerTest” packages [67] in R (version 3.4.3). The permutational multivariate analysis of variance (PERMANOVA) [68] was performed to assess the effects of fertilization and crop season on soil microbiome community structures through the adonis function with 999 permutations in the “vegan” package in R (version 3.4.3). The diversity (Shannon index) and community structure (PCoA1) of bacteria, fungi, and protists were selected as the six main microbial predictors, and the significance of effects of microbial predictors on crop yield was calculated using multiple regression by linear models in R (version 3.4.4). The analysis of the relative importance of the microbial predictors was run using the “relaimpo” package [69] in R (version 3.4.3). Analysis of similarity (ANOSIM) was performed to evaluate significant differences in microbial community structures across the four fertilizer treatments through the “anosim” command in Mothur [70]. As the community composition of protists was strongly affected by fertilization and was the only microbial group significantly linked with crop yield, we focused subsequent analyses on the protistan community. For that, One-way ANOVA with Tukey’s HSD test was performed to determine significant differences between treatments, and regression models were fitted in SPSS v20.0 (SPSS Inc. USA). Redundancy analysis (RDA) was performed to examine the relationships between abiotic factors (environmental variables) and the functional composition of protistan communities in the “vegan” package in R (version 3.4.3). Observed differences were tested for significance using the “envfit” function with 999 permutations. Further analyses focused on microbe-consuming protists as the only functional group showing significant correlations with crop yield. We used Spearman’s correlation coefficient to evaluate the correlation between abundant microbe-consuming protistan OTUs (top 50) and crop yield, abundant bacterial OTUs (average relative abundance > 0.5%), and abundant fungal OTUs (average relative abundance > 0.5%), respectively. Heat map analysis of the microbe-consuming protistan OTUs linked to crop yield across all treatments was carried out with the “pheatmap” package in R (version 3.4.3). All Spearman’s correlation coefficients were calculated with the “corr.test” function in the “psych” package in R (version 3.4.3). The *P* values were adjusted using the false discovery rate method [71]. Normal distribution was tested by the Shapiro-Wilk test, and non-normal data was log transformed [72].

Structural equation models were constructed to quantify links between crop yield and abundances of microbe-consuming protists, bacteria, and fungi. All variables were standardized by *Z* transformation (mean = 0, standard deviation = 1) to improve normality using the scale function in R [32]. The SEM construction and analysis were run using AMOS 17.0 (SPSS, Chicago, IL, USA). The covariance matrix was fit into the model using maximum likelihood estimation. The following metrices were used to ensure model fitting to the data: Chi-square (a model fits a given dataset well when *χ*^2^ is low), goodness-of-fit-index (GFI > 0.90), and root mean square error of approximation (RMSEA < 0.05) [73]. The fold change of plant biomass in different microbe-consuming protists inoculation relative to the control in pot experiment was calculated using the following formula: (*X* − Control)/Control, in which *X* is the plant biomass in different treatments with inoculation of different microbe-consuming protists, and Control represents the plant biomass in treatment without inoculation of microbe-consuming protists.

## Supplementary Information

**Additional file 1: Fig. S1.** Effects of different fertilization managements on cucumber yield across six cropping seasons (a). Protistan community based on unweighted unifrac distance of different fertilization managements across six cropping seasons (b). Effects of different fertilization managements (c) and crop seasons (d) on protistan diversity. Effects of different fertilization managements (e) and crop seasons (f) on microbe-consuming protists. **Fig. S2.** Redundancy analysis of the relationship between environmental variables and protistan functional groups (a). The relative importance of soil physicochemical properties for protistan diversity (b) and community structure (c). Heatmap illustrating the relationship between microbe-consuming protistan OTUs that are positively associated with crop yield and bacterial OTUs or fungal OTUs (relative abundance > 0.5%) in all treatments (d). **Fig. S3.** Fold change increase of cucumber biomass relative to the control in treatments with inoculation of two cercozoan species in the confirmatory second greenhouse experiment. **Table S1.** Fertilization scheme for chemical fertilizer (CF), organic fertilizer (OF) and bio-organic fertilizer (BF) in this study. **Table S2.** The effects of fertilization and crop season on distinct microbial groups based on PERMANOVA analysis. **Table S3.** The effects of fertilization and crop season on distinct microbial groups based on linear mixed model (LMM). **Table S4.** Spearman’ s rank correlation coefficient between cucumber yield and functional groups. **Table S5.** Soil physicochemical properties of different treatments in continuous cropping. **Table S6.** Detailed information of indicator protistan taxa (OTUs) based on PR2 database and GenBank.

## Data Availability

All raw sequence data have been made available in the NCBI Sequence Read Archive (SRA) database under the BioProject PRJNA599073.
