# Supplementary material for: Protists as main indicators and determinants of plant performance
Source: Microbiome. 2021 Mar 20;9:64. doi: 10.1186/s40168-021-01025-w (PMC7981826; doi:10.1186/s40168-021-01025-w)
Supplement: Supplementary file 2 — Additional file 1: Fig. S1. Effects of different fertilization managements on cucumber yield across six cropping seasons (a). Protistan community based on unweighted unifrac distance of different fertilization managements across six cropping seasons (b). Effects of different fertilization managements (c) and crop seasons (d) on protistan diversity. Effects of different fertilization managements (e) and crop seasons (f) on microbe-consuming protists. Fig. S2. Redundancy analysis of the relationship between environmental variables and protistan functional groups (a). The relative importance of soil physicochemical properties for protistan diversity (b) and community structure (c). Heatmap illustrating the relationship between microbe-consuming protistan OTUs that are positively associated with crop yield and bacterial OTUs or fungal OTUs (relative abundance > 0.5%) in all treatments (d). Fig. S3. Fold change increase of cucumber biomass relative to the control in treatments with inoculation of two cercozoan species in the confirmatory second greenhouse experiment. Table S1. Fertilization scheme for chemical fertilizer (CF), organic fertilizer (OF) and bio-organic fertilizer (BF) in this study. Table S2. The effects of fertilization and crop season on distinct microbial groups based on PERMANOVA analysis. Table S3. The effects of fertilization and crop season on distinct microbial groups based on linear mixed model (LMM). Table S4. Spearman’ s rank correlation coefficient between cucumber yield and functional groups. Table S5. Soil physicochemical properties of different treatments in continuous cropping. Table S6. Detailed information of indicator protistan taxa (OTUs) based on PR2 database and GenBank. [file 40168_2021_1025_MOESM2_ESM.docx]

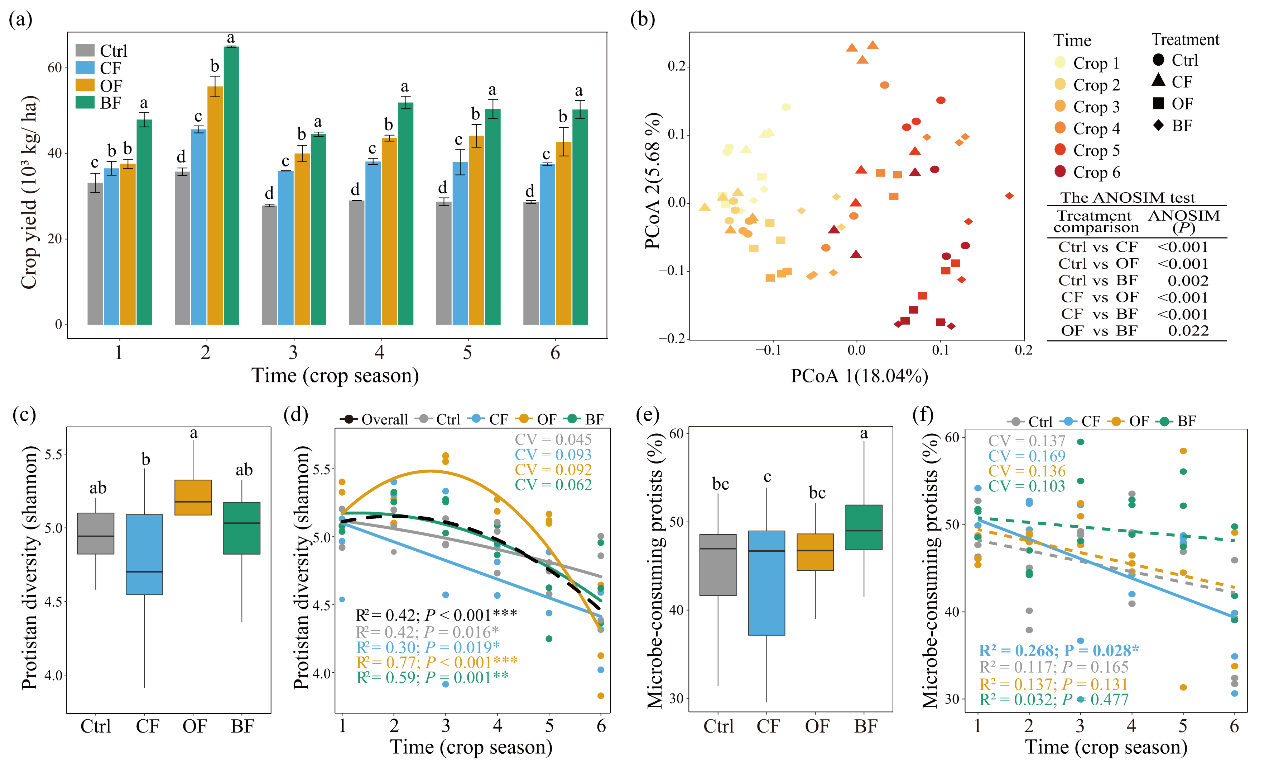


**Fig. S1. Effects of different fertilization managements on cucumber yield across six cropping seasons (a). Protistan community based on unweighted unifrac distance of different fertilization managements across six cropping seasons (b). Effects of different fertilization managements (c) and crop seasons (d) on protistan diversity. Effects of different fertilization managements (e) and crop seasons (f) on microbe-consuming protists.**

In panels (a), (c) and (e), bars with different letters indicate significant differences as defined by Tukey's HSD test (*P* < 0.05). In panels (d) and (f), ***indicates *P* < 0.001, *indicates *P* < 0.05. Statistical significances were calculated by regression analysis. Solid lines denote *P* < 0.05 and dotted lines denote *P* > 0.05. The coefficient of variability (CV) is standard deviation divided by the mean. In all panels, Ctrl: soil without fertilizer amendment; CF: chemical fertilizer addition; OF: organic fertilizer addition; BF: bio-organic fertilizer addition.


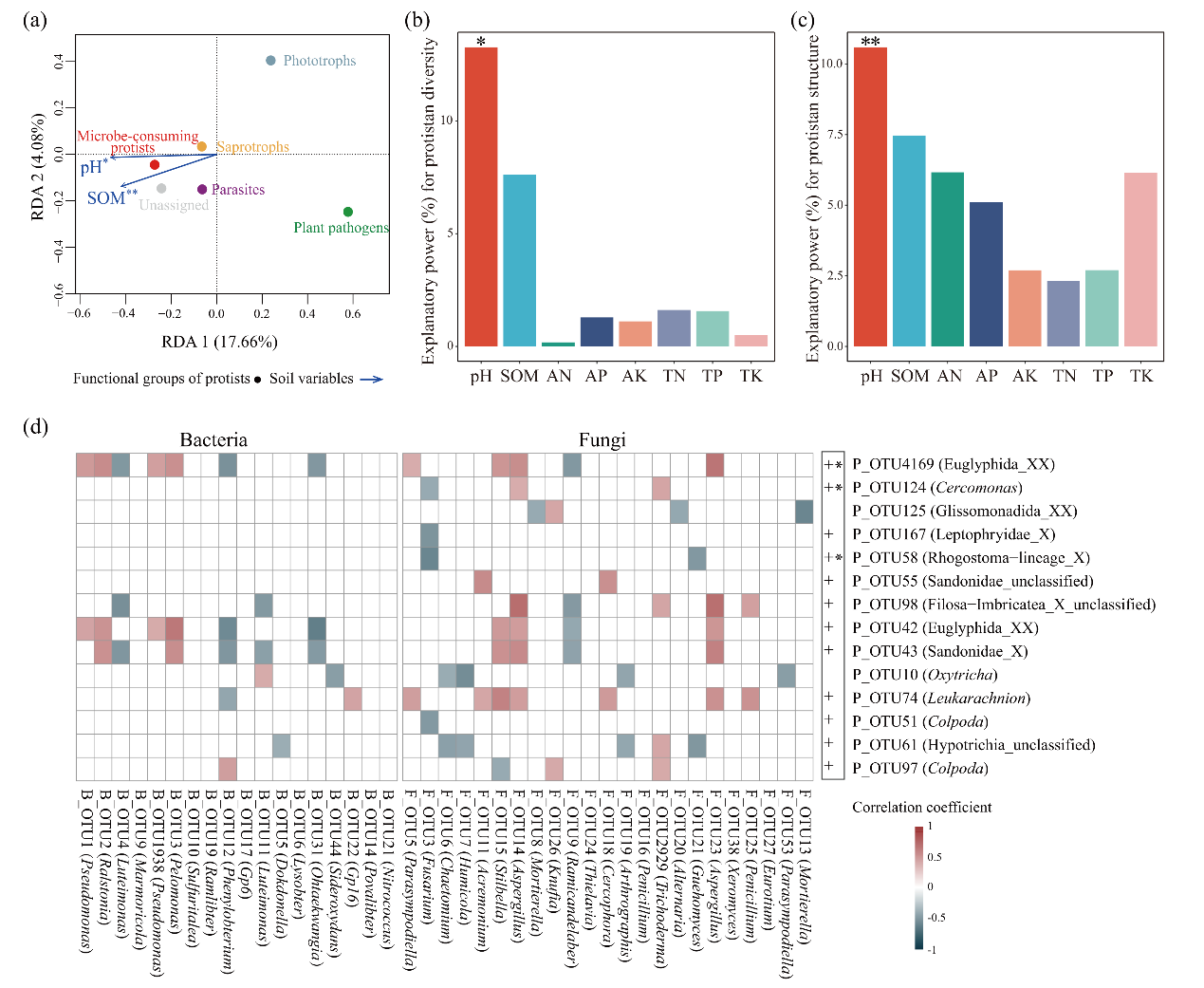


**Fig. S2. Redundancy analysis of the relationship between environmental variables and protistan functional groups (a). The relative importance of soil physicochemical properties for protistan diversity (b) and community structure (c). Heatmap illustrating the relationship between microbe-consuming protistan OTUs that are positively associated with crop yield and bacterial OTUs or fungal OTUs (relative abundance > 0.5%) in all treatments (d).**

In panels (a), **indicates *P* < 0.01, *indicates *P* < 0.05. Significance was tested using the “envfit” function using 999 permutations. Only significant soil variables (*P* < 0.05) were shown. In panels (b) and (c), **indicates *P* < 0.01, *indicates *P* < 0.05. Statistical significances were calculated by regression analysis. In panels (d), plus signs indicate higher relative abundance in OF and BF than in Control and CF, *indicates significantly higher relative abundance in OF and BF than in Control and CF, as defined by Tukey's HSD test (*P* < 0.05). Only significant correlations (*P* < 0.05) were shown in the figure.


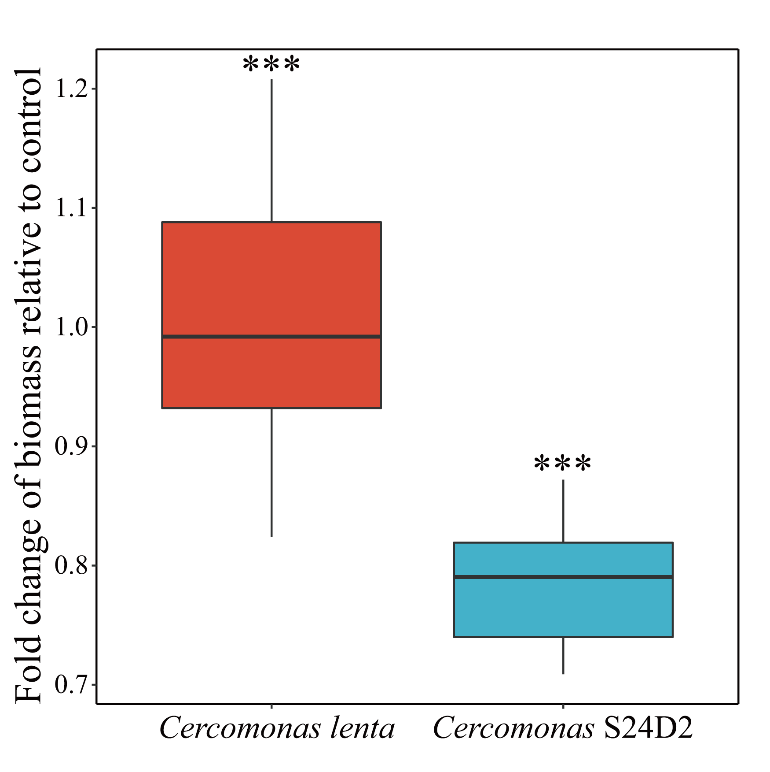


**Fig. S3. Fold change increase of cucumber biomass relative to the control in treatments with inoculation of two cercozoan species in** **the confirmatory second greenhouse experiment.**

Control indicates that no protists were inoculated. Asterisks indicate significant difference of cucumber biomass in treatments compared to control (Student’s t-test, ***indicates *P* < 0.001).

**Table S1. Fertilization scheme for chemical fertilizer (CF), organic fertilizer (OF) and bio-organic fertilizer (BF) in this study.**

|  | Fertilizers | Organic addition (kg/ha) | Inorganic N (kg/ha) | Inorganic P  (kg/ha) | Inorganic K (kg/ha) | Total N (kg/ha) | Total P (kg/ha) | Total K  (kg/ha) |
| --- | --- | --- | --- | --- | --- | --- | --- | --- |
| Basal fertilizer | CF | 0 | 120 | 120 | 120 | 120 | 120 | 120 |
|  | OF | 6000 | 54 | 17 | 34 | 120 | 120 | 120 |
|  | BF | 6000 | 54 | 17 | 34 | 120 | 120 | 120 |
| Supplementary fertilizer | CF | 0 | 60 | 0 | 60 | 60 | 0 | 60 |
|  | OF | 0 | 60 | 0 | 60 | 60 | 0 | 60 |
|  | BF | 0 | 60 | 0 | 60 | 60 | 0 | 60 |

Fertilization management programs: chemical fertilizer (CF), organic matter fertilizer (OF) and bio-fertilizer (BF).

**Table S2. The effects of fertilization and crop season on distinct microbial groups based on PERMANOVA analysis.**

|  |  | Fertilization | Crop season |
| --- | --- | --- | --- |
| Protistan community | R^2^ | 0.135 | 0.374 |
|  | *P* | 0.001 | 0.001 |
| Bacterial community | R^2^ | 0.111 | 0.362 |
|  | *P* | 0.001 | 0.001 |
| Fungal community | R^2^ | 0.123 | 0.367 |
|  | *P* | 0.001 | 0.001 |

**Table S3. The effects of fertilization and crop season on distinct microbial groups based on linear mixed model (LMM).**

|  |  | Fertilization | Crop season |
| --- | --- | --- | --- |
| Protistan alpha diversity | F | 3.944 | 39.844 |
|  | *P* | 0.053 | 0.001 |
| Bacterial alpha diversity | F | 0.881 | 7.580 |
|  | *P* | 0.491 | 0.008 |
| Fungal alpha diversity | F | 2.166 | 1.107 |
|  | *P* | 0.170 | 0.297 |

With the plot position serial number (i _ j, are the row and column number of the plot, respectively) in the field as a random effect.

**Table S4. Spearman's rank correlation coefficient between cucumber yield and functional groups.**

|  |  | | Cucumber yield | |
| --- | --- | --- | --- | --- |
| Functional groups | | Microbe-consuming protists | | **0.24** |
|  |  | Parasites | | NS |
|  |  | Phototrophs | | NS |
|  |  | Plant pathogens | | NS |
|  |  | Saprotrophs | | NS |
|  |  | Unassigned function | | NS |

­ NS = not significant (significant difference at the 0.05 probability level).

**Table S5. Soil physicochemical properties of different treatments in continuous cropping.**

| Property | Ctrl | CF | OF | BF |
| --- | --- | --- | --- | --- |
| pH | 7.19±0.07 a | 7.06±0.22 b | 7.29±0.09 a | 7.20±0.05 a |
| Organic Matter (g/kg) | 24.23±1.33 b | 23.25±1.77 b | 26.60±0.85 a | 26.95±1.31 a |
| Available N (mg/kg) | 250.25±31.59 c | 351.74±53.52 a | 304.01±31.79 b | 281.80±51.71 bc |
| Total N (g/kg) | 1.53±0.09 c | 1.61±0.14 c | 1.86±0.08 a | 1.71±0.07 b |
| Available P_2_O_5_ (mg/kg) | 215.53±17.40 c | 241.32±9.89 b | 257.90±8.28 a | 259.81±8.45 a |
| Total P_2_O_5_ (g/kg) | 0.99±0.07 b | 1.05±0.08 b | 1.14±0.05 a | 1.13±0.05 a |
| Available K_2_O (mg/kg) | 322.47±12.40 c | 328.56±11.51 c | 353.42±14.21 b | 366.53±11.58 a |
| Total K_2_O (g/kg) | 4.44±0.22 d | 5.47±0.23 a | 5.16±0.20 b | 4.67±0.34 c |

Different letters indicate significant differences at *P* < 0.05 according to Tukey's HSD test. Fertilization management programs: no fertilizer (Ctrl), chemical fertilizer (CF), organic fertilizer (OF) and bio-organic fertilizer (BF).

**Table S6. Detailed information of indicator protistan taxa (OTUs) based on PR2 database and GenBank**.

| OTUid | Taxonomy annotation (PR2) | Taxonomy annotation (GenBank) | Sequence ID (GenBank) | The percentage of similarity (GenBank) |
| --- | --- | --- | --- | --- |
| OTU124 | Eukaryota;Rhizaria;Cercozoa;Filosa-Sarcomonadea;Cercomonadida;Cercomonadidae;  Cercomonas;Cercomonas_sp.; | *Cercomonas* sp. | KY974770.1 | 100% |
| OTU4169 | Eukaryota;Rhizaria;Cercozoa;Filosa-Imbricatea;Euglyphida;Euglyphida_X;Euglyphida_XX;Euglyphida_XX_sp.; | *Euglyphida* sp. | KY975190.1 | 98% |
| OTU58 | Eukaryota;Rhizaria;Cercozoa;Filosa-Thecofilosea;Cryomonadida;Rhogostoma-lineage;  Rhogostoma-lineage_X;Rhogostoma-lineage_X_sp.; | *Rhogostoma schuessleri* | HQ121430.1 | 96% |
| OTU51 | Eukaryota;Alveolata;Ciliophora;Colpodea;Colpodida;Colpodidae;Colpoda;Colpoda_unclassified; | *Colpoda* sp*.* | MK801290.1 | 99% |
| OTU97 | Eukaryota;Alveolata;Ciliophora;Colpodea;Colpodida;Colpodidae;Colpoda;Colpoda_steinii; | *Colpoda steinii* | KJ607912.1 | 99% |
| OTU74 | Eukaryota;Stramenopiles;Ochrophyta;Chrysophyceae;Chrysophyceae_X;Chrysophyceae_XX;Leukarachnion;Leukarachnion_sp.; | *Leukarachnion* sp*.* | FJ356265.2 | 99% |
| OTU67 | Eukaryota;Stramenopiles;Ochrophyta;Chrysophyceae;Chrysophyceae_X;Chrysophyceae_Clade-C;Spumella;Spumella_sp.; | *Spumella* sp. | MN945084.1 | 99% |
| OTU121 | Eukaryota;Amoebozoa;Conosa;Variosea;ATCC50593-Flamella-WIM80-lineage;Flamella-lineage;Flamella;Flamella_sp.; | *Flamella aegyptia* | EU186021.1 | 99% |
| OTU1450 | Eukaryota;Amoebozoa;Lobosa;Tubulinea;Echinamoebida;Vermamoebidae;Vermamoebidae_unclassified;Vermamoebidae_unclassified; | *Vermamoeba vermiformis* | KU519742.1 | 99% |
| OTU22 | Eukaryota;Alveolata;Ciliophora;Spirotrichea;Hypotrichia;Pseudourostylidae;Pseudourostyla;Pseudourostyla_franzi; | *Pseudourostyla franzi* | AM412765.1 | 99% |
| OTU167 | Eukaryota;Rhizaria;Cercozoa;Endomyxa;Vampyrellida;Leptophryidae;Leptophryidae_X;Leptophryidae_X_sp.; | *Leptophryidae* sp. | KC779513.1 | 98% |
| OTU10 | Eukaryota;Alveolata;Ciliophora;Spirotrichea;Hypotrichia;Oxytrichidae;Oxytricha;Oxytricha_sp.; | *Oxytricha lithofera* | MT364897.1 | 98% |
| OTU45 | Eukaryota;Amoebozoa;Lobosa;Tubulinea;Echinamoebida;Vermamoebidae;Hartmannella;Hartmannella_vermiformis; | *Hartmannella vermiformis* | DQ123623.2 | 98% |
| OTU42 | Eukaryota;Rhizaria;Cercozoa;Filosa-Imbricatea;Euglyphida;Euglyphida_X;Euglyphida_XX;Euglyphida_XX_sp.; | *Euglyphida* sp. | KY975190.1 | 97% |
| OTU55 | Eukaryota;Rhizaria;Cercozoa;Filosa-Sarcomonadea;Glissomonadida;Sandonidae;Sandonidae_unclassified;Sandonidae_unclassified; | *Bodomorpha* sp. | HM536172.1 | 97% |
| OTU125 | Eukaryota;Rhizaria;Cercozoa;Filosa-Sarcomonadea;Glissomonadida;Glissomonadida_X;Glissomonadida_XX;Glissomonadida_XX_sp.; | *Kraken* sp. | KY976683.1 | 97% |
| OTU61 | Eukaryota;Alveolata;Ciliophora;Spirotrichea;Hypotrichia;Hypotrichia_unclassified;Hypotrichia_unclassified;Hypotrichia_unclassified; | *Stichotrichia* sp. | LN870140.1 | 95% |
| OTU98 | Eukaryota;Rhizaria;Cercozoa;Filosa-Imbricatea;Filosa-Imbricatea_X;Filosa-Imbricatea_X_unclassified;Filosa-Imbricatea_X_unclassified;Filosa-Imbricatea_X_unclassified; | *Nudifila producta* | HQ121434.1 | 95% |
| OTU43 | Eukaryota;Rhizaria;Cercozoa;Filosa-Sarcomonadea;Glissomonadida;Sandonidae;Sandonidae_X;Sandonidae_X_sp.; | *Bodomorpha* sp. | DQ211595.1 | 95% |
